# Alternating bipolar field stimulation identifies muscle fibers with defective excitability but maintained local Ca^2+^ signals and contraction

**DOI:** 10.1186/s13395-016-0076-8

**Published:** 2016-02-05

**Authors:** Erick O. Hernández-Ochoa, Camilo Vanegas, Shama R. Iyer, Richard M. Lovering, Martin F. Schneider

**Affiliations:** 1 Department of Biochemistry and Molecular Biology, School of Medicine, University of Maryland, Baltimore, 108 N. Greene Street, Baltimore, MD 21201 USA; 2 Department of Orthopaedics, University of Maryland School of Medicine, Baltimore, MD 21201 USA

**Keywords:** Skeletal muscle, Excitation-contraction coupling, Enzymatic dissociation, Cultured myofibers, Abnormal excitability

## Abstract

**Background:**

Most cultured enzymatically dissociated adult myofibers exhibit spatially uniform (UNI) contractile responses and Ca^2+^ transients over the entire myofiber in response to electric field stimuli of either polarity applied via bipolar electrodes. However, some myofibers only exhibit contraction and Ca^2+^ transients at alternating (ALT) ends in response to alternating polarity field stimulation. Here, we present for the first time the methodology for identification of ALT myofibers in primary cultures and isolated muscles, as well as a study of their electrophysiological properties.

**Results:**

We used high-speed confocal microscopic Ca^2+^ imaging, electric field stimulation, microelectrode recordings, immunostaining, and confocal microscopy to characterize the properties of action potential-induced Ca^2+^ transients, contractility, resting membrane potential, and staining of T-tubule voltage-gated Na^+^ channel distribution applied to cultured adult myofibers. Here, we show for the first time, with high temporal and spatial resolution, that normal control myofibers with UNI responses can be converted to ALT response myofibers by TTX addition or by removal of Na^+^ from the bathing medium, with reappearance of the UNI response on return of Na^+^. Our results suggest disrupted excitability as the cause of ALT behavior and indicate that the ALT response is due to local depolarization-induced Ca^2+^ release, whereas the UNI response is triggered by action potential propagation over the entire myofiber. Consistent with this interpretation, local depolarizing monopolar stimuli give uniform (propagated) responses in UNI myofibers, but only local responses at the electrode in ALT myofibers. The ALT responses in electrically inexcitable myofibers are consistent with expectations of current spread between bipolar stimulating electrodes, entering (hyperpolarizing) one end of a myofiber and leaving (depolarizing) the other end of the myofiber. ALT responses were also detected in some myofibers within intact isolated whole muscles from wild-type and MDX mice, demonstrating that ALT responses can be present before enzymatic dissociation.

**Conclusions:**

We suggest that checking for ALT myofiber responsiveness by looking at the end of a myofiber during alternating polarity stimuli provides a test for compromised excitability of myofibers, and could be used to identify inexcitable, damaged or diseased myofibers by ALT behavior in healthy and diseased muscle.

**Electronic supplementary material:**

The online version of this article (doi:10.1186/s13395-016-0076-8) contains supplementary material, which is available to authorized users.

## Background

Numerous research groups have studied many cellular aspects of muscle function and disease using individual myofibers isolated from hind-limb muscles, including the extensor digitorum longus (EDL), flexor digitorum brevis (FDB), dorsal interosseous, lumbricals and soleus. These muscles are anatomically located at points of relatively easy accessibility, and their gross dissection is not complicated. Once dissected, individual muscle myofibers are obtained either via a delicate manual single myofiber dissection, a method known as the intact myofiber preparation [1], or through the use of enzymatic and mild mechanical dissociation techniques [2]. Each technique has advantages and disadvantages. The single intact myofiber preparation has exceptional advantages for studies where accurate measurements and changes of sarcomere length and fiber-specific force are required [1, 3], and the tendon is needed for mechanical attachment. However, this technique requires high dissection skills and is difficult to learn [1, 4]. In contrast, enzymatic dissociation is a relatively less complicated procedure that can yield hundreds of fibers, so enzymatically dissociated muscle myofibers have been increasingly used in cellular studies of skeletal muscle. Early studies of dissociated FDB myofibers evaluated the morphological and electrophysiological properties of myofibers from different models [2, 5–8]. Since then, numerous laboratories have used dissociated FDB myofibers in studies of the membranous system and contractile apparatus [9–11] and in other areas such as excitation-contraction coupling (ECC) and Ca^2+^ homeostasis [11–32], metabolism [33, 34], cellular signaling, and gene regulation [16, 35–56].

Although the unique advantages of the enzymatically dissociated FDB muscle myofibers provided valuable information and proved useful to characterize numerous aspects of muscle function at the cellular level for the past three decades [2, 57–62], the technique may have some disadvantages.

We recently reported the presence of a subgroup of FDB muscle myofibers that exhibit local alternating end contraction and Ca^2+^ transients upon alternating polarity electric field stimulation by remote bipolar electrodes, from here on referred as to ALT myofibers [63]. The existence of ALT myofibers in primary cultures has been shown by us to increase in myofiber cultures overexpressing the transcription factor Foxo1 [46]. However, a detailed study of the electrophysiological properties of ALT myofibers, to the best of our knowledge, has not been carried out. Here, we present methodology for the study of the excitable properties of the ALT myofibers evaluated with bipolar field stimulation, and propose a potential mechanism to account for these unusual local Ca^2+^ responses. One additional goal was also to compare monopolar focal stimulation with the more commonly used bipolar field stimulation. Finally, besides characterizing the spatio-temporal details of electrically evoked Ca^2+^ transients and underlying mechanisms in cultured myofibers, we also wanted to determine whether ALT behavior could be observed in whole muscle before enzymatic dissociation. Importantly, in intact whole muscles from healthy and MDX mice loaded with Ca^2+^ indicator, we were able to detect ALT fibers in response to bipolar field stimulation in some fibers within the whole muscle before any fiber dissociation. We propose that checking for the presence of the ALT response may provide a tool for detecting fibers with compromised excitability due to muscle disease or other factors. A preliminary account of some of these results has been presented at the Biophysical Society Meeting [63].

## Methods

### Ethical approval

All animals were housed in a pathogen-free area at the University of Maryland, Baltimore. The animals were euthanized according to authorized procedures of the Institutional Animal Care and Use Committee, University of Maryland, Baltimore, by regulated delivery of compressed CO_2_ overdose followed by cervical dislocation.

### Model system

Six- to eight-week-old male C57BL/6 N mice were euthanized, and the flexor digitorum brevis (FDB) muscles were harvested bilaterally. A total of 8–10 mice were used (approximately 4–8 weeks of age). For whole muscle experiments, we used male control mice (wild-type, WT) and MDX mice (lacking dystrophin), both from the C57BL/10ScSnJ strain (The Jackson Laboratory, Bar Harbor, ME). A total of six mice were used (approximately 4–8 weeks of age). Following euthanasia, flexor digitorum brevis (FDB) and extensor digitorum longus (EDL) muscles were harvested bilaterally from MDX and WT mice. Single myofibers from FDB muscles were enzymatically isolated in MEM with 1 μl/ml Gentamicin (Sigma, St. Louis, MO; Cat. No. G1397) and 2 mg/ml type I collagenase (Sigma, Cat. No. C0130) for 3–5 h at 37 °C as previously described [32, 45]. Solutions were filtered using a 0.2-μm polyethersulfone membrane (www.thermoscientific.com, Cat. No. 90–9920). Myofibers were then plated on glass-bottomed dishes (Matek Cor. Ashland, MA, Cat. No. P35G-1.0-14-C,) coated with laminin (Thermo Fisher, Rockford, IL, Cat. No. 23017–015) and cultured (5 % CO2; 37 °C) for 12–18 h before experiments.

### Transverse tubular network imaging in living myofibers

Myofibers were stained with the voltage-sensitive dye pyridinium, 4-[2-(6-(dioctylamino)-2-naphthalenyl) ethenyl]-1-(3-sulfopropyl)-, inner salt (di-8-ANEPPS; Thermo Fisher, Cat. No. D3167) as previously described [45, 52]. Briefly, myofibers were incubated with di-8-ANEPPS 2.5 μM/L in MEM media for 2- 4 h at 37 °C and then imaged on a Fluoview 500 confocal system (Olympus, Waltham, MA); using a × 60, 1.3 NA water-immersion objective using L-15 media (ionic composition in mM: 137 NaCl, 5.7 KCl, 1.26 CaCl_2_, 1.8 MgCl_2_, pH 7.4; Thermo Fisher, Cat. No.21083-027) as recording solution. Confocal images (512 × 512 pixels) of the tubular network were obtained from randomly selected myofibers using the same image acquisition settings and enhancing parameters. Images were background corrected, and a region of interest of fixed dimensions was used to estimate average fluorescence profile within the region of interest.

### Ca^2+^ imaging

*Dissociated myofiber Ca* ^*2+*^ *imaging*. Rhod-2 measurements were carried out on a high-speed confocal system (LSM 5 Live system, Carl Zeiss, Jena, Germany) as previously described [46, 64]. Briefly, myofibers were loaded with 1 μM rhod-2 AM (Thermo Fisher, Cat. No. R-1245MP) in L-15 media supplemented with 0.25 % w/v bovine serum albumin (L-15BSA) for 1 h at room temperature. Individual myofibers were imaged with either a × 10/0.3 NA or a × 60/1.3 NA water-immersion objective lens using L-15 as recording solution. Where indicated, a Na^+^-free or low [Cl^−^] extracellular recording solution was used to evaluate the effects of Na^+^ and Cl^−^ extracellular concentration, on electrically evoked Ca^2+^ signals in UNI and ALT fibers. The Na^+^-free solution contained (in mM): 150 NMDG, 10 HEPES, 2 CaCl_2_, 1 MgCl_2_, pH adjusted to 7.4 with CsOH. The low [Cl^−^] solution contained (in mM): 150 Na-CH_3_SO_3_, 10 HEPES, 2 CaCl_2_, 1 MgCl_2_, pH adjusted to 7.4 with NaOH. Excitation for rhod-2 was provided by the 532-nm line of a 100-mW diode laser, and emitted light was collected at >550 nm. The confocal imaging was conducted at a depth of ∼ 20 μm into the interior of the myofiber. In the majority of the experiments, 0.5-1 ms electrical field stimuli were applied via two parallel platinum wires positioned perpendicular to the bottom of the dish, ∼5 mm apart, to elicit action potentials (myofibers were centrally positioned at less than about a ± 45° angle relative to an imaginary line between the tips of the electrodes). In a few experiments (where noted), a unipolar electrode was positioned about ∼ 100 μm away from the myofiber surface, either near the center or near the end the myofiber. Application of each stimulation protocol was synchronized relative to the start of confocal scan acquisition. Typically, the field stimulus was applied 100 ms after the start of the confocal scan sequence, thus providing control images before stimulation at the start of each sequence. These control images were used to determine the resting steady-state fluorescence level (F0). Average intensity of fluorescence within selected regions of interest (ROIs) within a myofiber was measured with Zeiss LSM Image Examiner (Carl Zeiss, Jena, Germany). Images in line-scan (*xt*) mode (frame size: 512 × 10,000 pixels; scan speed: 100 μs/line for 1 s acquisition) or field imaging (*xy*) mode (frame size: 512 × 512) at 60 frames/s were background corrected by subtracting an average value recorded outside the cell. The average F0 value in each ROI before electrical stimulation was used to scale Ca^2+^ signals in the same ROI as ΔF/F0. No attempts were made to estimate the actual cytosolic Ca^2+^ concentration. Ca^2+^ imaging experiments were carried out at room temperature, 21–23 °C. *Whole muscle Ca* ^*2+*^ *imaging*; FDB and EDL muscles were carefully dissected, keeping tendons at both extremities. After loading for 30 min with fluo-4-AM (Thermo Fisher, Cat. No. F-14201) in L-15BSA, the muscle was held in place with a plastic coverslip attached with vacuum grease in a petri dish (see Additional file 1: Figure S3) and stimulated with a single pulse followed by a train of four pulses (1-ms pulse duration) at 5 Hz, with all pulses alternating in polarity. Imaging was performed with the same apparatus as used in rhod-2 measurements. Excitation for fluo-4 was provided by the 488-nm line of a 100-mW diode laser, and emitted light was collected at >505 nm. Line scanning (512 x 2000 lines) was collected at 2 ms/line. Images are presented as F/F_0_.

### Resting Ca^2+^ measurements

Indo-1 acetoxymethyl (AM) ratiometric imaging and analysis were performed as previously described [46]. Briefly, cultured FDB fibers were loaded with indo-1 AM (1 μM/L for 60 min at 22 °C; Thermo Fisher, Cat. No. I-1223) in L-15 media. The culture dish was mounted on an Olympus IX71 inverted microscope and viewed with an Olympus × 60/1.20 NA water-immersion objective. Fibers were illuminated at 360 nm, and the fluorescence emitted at 405/30 and 485/25 nm was detected simultaneously. The emission signals were digitized and sampled at 2 KHz using a built-in AD/DA converter of an EPC10 amplifier and the acquisition software Patchmaster (HEKA Instruments Inc., Bellmore, NY).

### Membrane potential measurements

Resting membrane potential was measured using a conventional microelectrode amplifier (AXOCLAMP-2; Axon Instruments Inc., USA). Microelectrodes were filled with 1 M K-acetate and resistances ranged between 12–40 MΩ, as previously described by others [7, 62].

### Immunocytofluorescence

Immunostaining was performed according to previously published methods [45]. Myofibers were fixed in PBS (pH 7.4) containing 4 % (w/v) of paraformaldehyde for 20 min, permeabilized in PBS containing 0.1 % (v/v) Triton × 100 (Sigma) for 15 min, exposed to PBS containing 8 % (v/v) of goat serum for 1 h at room temperature, 21–23 °C, to block non-specific labeling, and then incubated with a primary rabbit antibody against the II-III intracellular loop residues 877–891 of Na_v_1.4 (1:100, overnight at 4 °C; a kind gift from Alomone Labs, Jerusalem, Israel, Cat No. ASC-020), followed by incubation with Alexa488-conjugated goat anti-rabbit antibody (1:200 dilution, 2 h at RT; Thermo Fisher, Cat. No. A-11034). To test for the specificity of the primary antibody, in some dishes the primary antibody was preincubated with a Na_v_1.4_877–891_ neutralizing peptide (Alomone Labs, Cat No. ASC-020) before labeling and used as a negative control. Antibody-labeled myofibers and antibody/peptide incubated myofibers were imaged on a Fluoview 500 Olympus LSM system, based on an IX/71 inverted microscope using a × 60 NA 1.2 water-immersion objective lens. The excitation for alexa-488 was provided by using a 488-nm laser. The emitted light for alexa-488 was collected at >510 nm. Prior fiber fixation, a computer-controlled microscope stage and field stimulation, mounted in our Fluoview 500 Olympus LSM system, were used to select, identify, and save in a file the locations of numerous ALT and UNI fibers in multiple dishes. After the immunostaining, we used the same LSM image system and computer-controlled stage to acquire unequivocally fluorescence confocal images from fibers previously identified as UNI or ALT. Confocal images of UNI and ALT myofibers were collected using the same image acquisition settings and enhancing parameters so that all images can be directly compared. Images were background corrected. Images were processed using ImageJ (NIH, Bethesda, MD, USA; http://rsb.info.nih.gov/ij/).

### Data analysis

All data processing and statistical analysis was performed using OriginPro 8.0. All data are presented as mean ± SEM unless otherwise noted. Statistical significance was assessed using either parametric two-sample *t* test or with the non-parametric Mann-Whitney rank-sum test. Significance was set at *P* < 0.05.

## Results

### Alternating end local contractions in a subpopulation of FDB myofibers in response to electrical field stimuli of alternating polarity

Using light microscopy and simultaneously monitoring the myofiber twitch activity in response to brief suprathreshold electrical field stimulation of alternate polarity, it is possible to distinguish three types of myofiber responses [46]: (1) the vast majority of the myofibers display a “normal” uniform (UNI) behavior (see video in Additional file 2, *left*); these myofibers exhibit a symmetric twitch contraction at both ends and concentric myofiber shortening in response to a brief (0.5–1 ms; 15 V/cm) external field stimuli of alternating polarity, indicative of a propagated action potential and uniform contractile activation; (2) another subset of myofibers (5–20 %) responds differently; these myofibers exhibit local “asymmetric” contraction upon alternating polarity external stimulation (see video in Additional file 2, *right*); from here on referred as to ALT myofibers; (3) some myofibers do not twitch at all in response to electrical stimulation; these myofibers represent a variable but very small fraction of the overall myofiber population and were not studied in detail in the present work.

### Local electrically evoked Ca^2+^ transients in ALT myofibers in response to electrical field stimuli of alternating polarity

**Fig. 1:**
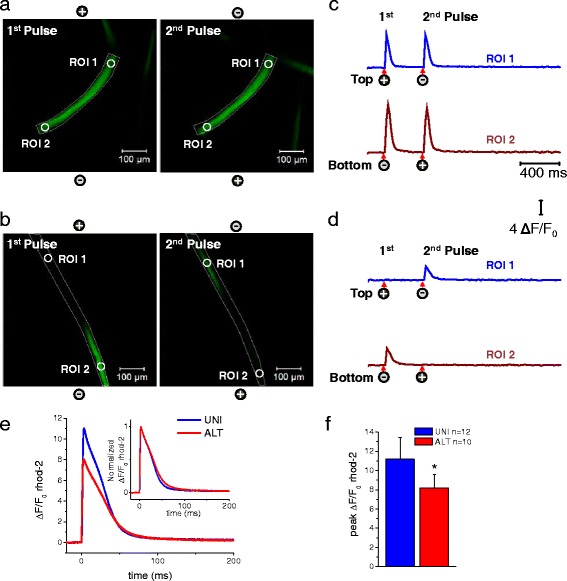
Electrically induced Ca^2+^ transients in myofibers exhibiting spatially uniform activation (UNI) or myofibers showing alternate end activation (ALT) in response to alternating polarity electric field stimulation. Spatio-temporal properties of depolarization-induced rhod-2 Ca^2+^ transients in myofibers that responded with uniform responses (**a** UNI) and myofibers showing local alternating end responses (**b** ALT) when stimulated with a bipolar field stimulation of alternating polarity and imaged in frame mode (xy-t; 60 frames/s) using high-speed confocal microscope. Images labeled as first and second in **a** and **b** represent snap shots of the time series at the peak of the transients in response to two sequential field pulses (0.5 ms; 15 V/cm) separated by an interval of 400 ms. See video in Additional file 3 for the entire time series. UNI myofibers exhibit a global Ca^2+^ transient in response to pulses of both polarities during alternating polarity stimulation, whereas ALT myofibers display only local non-propagated responses at alternating ends upon application of pulses of alternate polarity. The polarity signs indicate the location of the electrodes and their polarity for each stimulus. **c** and **d** are shown the time course of the rhod-2 fluorescence measured at the ends of the myofibers. *Circles* labeled as regions of interest (ROI) show ROI 1 (myofiber’s *upper end*, *blue trace*) and ROI 2 (myofiber’s *lower end*, *dark red trace*) show the locations used to measure the time course of the rhod-2 fluorescence. *Arrows* and *signs* under the traces indicate both the polarity and the time when the pulses where applied. Vertical scale: ΔF/F_0_ = 4; horizontal scale: time 400 ms. **e** Averaged time courses of the rhod-2 fluorescence measured in UNI myofibers and at the responding end of the ALT myofibers using ultra high-speed line scanning (100 μs/line) to further characterize the temporal properties of the Ca^2+^ transients. *Inset*, Time course of the normalized Ca^2+^ transients in the UNI and ALT myofibers derived from panel *E* to compare the kinetics of Ca^2+^ transients. **f** Bar plot summarizing differences in peak amplitude of rhod-2 ΔF/F0 transient. UNI fibers: ΔF/F0 = 11.18 ± 2.2, *n* = 12, *N* = 3; ALT fibers: ΔF/F0 = 8.15 ± 1.4, *n* = 10, *N* = 3. *N* indicates number of mice per condition, and *n* indicates number of fibers tested.*Indicates *P* < 0.05 when compared with UNI control fibers, two-sample *t* test

Proper electrical excitation and Ca^2+^ release is essential for muscle contraction and voluntary movements. To determine whether ALT myofibers exhibit deficits in the conformational coupling between the voltage sensors, the Ca_v_1.1 channels, and the RyR1, the Ca^2+^ release channels, we monitored electrically induced Ca^2+^ transients using high-speed confocal microscopy. When UNI myofibers are loaded with a fluorescent Ca^2+^ indicator, the myofibers display a global Ca^2+^ transient in response to external brief stimulation of both polarities, indicating proper excitation, Ca^2+^ release and contraction-relaxation cycle (Fig. 1a; see video in Additional file 3, *left*). However, when the ALT myofibers are loaded with a fluorescent Ca^2+^ indicator, the myofibers display only local non-propagated Ca^2+^ transients at alternating ends upon external stimulation with alternating polarity pulses (Fig. 1b; see video in Additional file 3, *right*). As quantified in Fig. 1e, f, ALT myofibers demonstrate a 27 % decrease in peak ΔF/F0 in the responding end of the myofiber when compared with UNI controls following single field electrical stimulation (UNI: ΔF/F0 = 11.18 ± 2.2, *n* = 12, *N* = 3; ALT: ΔF/F0 = 8.15 ± 1.4, *n* = 10, *N* = 3; *P* < 0.05). Thus, ALT fibers appear to have only local activation and disrupted action potential-induced Ca^2+^ transients. Consistent with this observation, the amplitude and duration of ALT, but not UNI responses, are graded with stimulus duration (see Additional file 1: Figure S1). The gross morphology of UNI and ALT myofibers was remarkably similar. Both myofibers exhibited smooth surfaces, clear striations, no bends and no signs of contractures. In addition, ALT fibers had resting indo-1 ratios that were not significantly different from UNI fibers, indicating that resting Ca^2+^ concentrations are not modified in ALT fibers (indo-1 ratio was 0.64 ± 0.04, *n* = 13, *N* = 3 in UNI fibers vs. 0.72 ± 0.04, *n* = 10, *N* = 3, in ALT fibers, *P* > 0.05). These observations raised several questions as to why these ALT myofibers respond the way they do to electrical field stimulation. Are ALT myofibers partially depolarized due to enzymatically and or trituration-induced membrane damage, which would explain the ALT behavior? Do they exhibit structural alterations in the T-tubule system, the network of extensions of the surface membrane responsible for propagation of the muscle electrical impulse radially into the myofiber?

### RMP in UNI and ALT fibers

One possibility that could account for the behavior of the ALT fibers in response to bipolar field stimulation is membrane damaged inflicted by enzymatic digestion and or trituration, which in turn could cause membrane depolarization and inexcitability. To test for this possibility, we measured resting membrane potential (RMP) using conventional microelectrodes in UNI and ALT fibers (see Additional file 1: Figure S2). To avoid Cl^−^-related membrane depolarization and obtain more stable records, we used K^+^-acetate filled electrodes [7, 62]. We found no significant difference in RMP measurements in previously identified dissociated UNI or ALT fibers (see Additional file 1: Figure S2).

### T-tubule and surface membrane structure of ALT myofibers compared with control counterparts

**Fig. 2:**
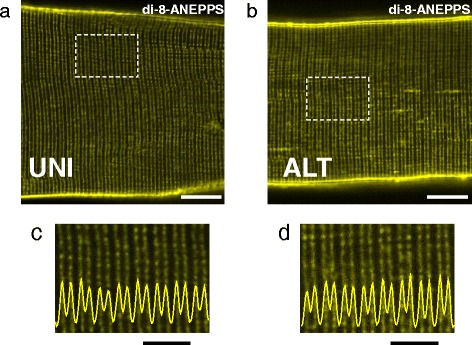
Evaluation of the T-tubule system of UNI and ALT myofibers. **a, b** Representative confocal images of a UNI myofiber (**a**) or an ALT myofiber (**b**) stained with di-8-ANEPPS to visualize the sarcolemma and T-tubules. *Scale bars* in **a, b** are 10 μm. **c, d** Bottom panels are zoom-in versions of boxed regions indicated in panels **a** and **b**. Traces inserted in zoomed-in images are averaged fluorescence profiles across the box, *scale bars*: 5 μm. In control UNI myofibers (*n* = 18, *N* = 3), T-tubules are organized in a regular striated pattern. No changes in T-tubule morphology are seen in ALT myofibers (*n* = 16, *N* = 3). *N* indicates number of mice per condition, and *n* indicates number of myofibers imaged

Efficient coupling between excitation and the Ca^2+^ release machinery in skeletal muscle requires both functional and structural intact triad junctional complexes comprised of T-tubule invaginations of plasma membrane and terminal cisternae of sarcoplasmic reticulum. The T-tubule system is the membrane system along which depolarization spreads inward into the myofiber [65, 66]. Breakdown of this system would disrupt propagation of the action potential and thus the contraction of the myofiber. Considering the important role of the T-tubule system in excitation-contraction coupling, we examined if changes in T-tubule morphology could account for the contractile and Ca^2+^ release deficits seen in the ALT myofibers. Di-8-ANEPPS, a lipophilic membrane dye, was used to image the surface and T-tubule membrane system. Typical T-tubule doublets bracketing the Z line were observed in both control (Fig. 2a, c) and ALT myofibers (Fig. 2b, d), suggesting that the gross morphology of the membrane system of the T-tubule network was unaltered in the ALT myofibers.

### Reduction of extracellular [Na^+^] or TTX treatment “converts” UNI myofibers to ALT myofibers

**Fig. 3:**
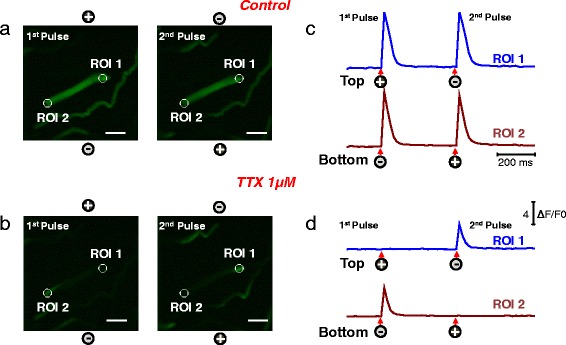
Myofibers exhibiting UNI responses under control conditions exhibit local Ca^2+^ transients (ALT responses) when treated with TTX. Myofibers with uniform responses were loaded with rhod-2, and their Ca^2+^ responses to electrical stimulation were recorded before and after the addition of TTX, a voltage-dependent Na^+^ channel blocker. Spatio-temporal properties of depolarization-induced rhod-2 Ca^2+^ transients in a myofiber that exhibited uniform responses in a physiological recording solution (**a** UNI) and ALT responses after treatment with TTX (**b**). Rhod-2 signals were imaged and analyzed as in Fig. 1 (see video in Additional file 4 for the entire time series). *Scale bars* in **a–b** are 100 μm. UNI myofibers exhibit a global Ca^2+^ transient in response to pulses of alternate polarity; however, the addition of TTX “converts” UNI myofibers into ALT myofibers that display only local non-propagated Ca^2+^ transients in response to pulses of alternate polarity, as the ALT myofibers described in Fig. 1b. The polarity signs indicate the location of the electrodes and their polarity for each stimulus. **c, d** show the time course of the rhod-2 fluorescence measured at the ends of the myofibers. *Circles* labeled as regions of interest (ROI) ROI 1 (myofiber’s *upper end*, *blue trace*) and ROI 2 (myofiber’s *lower end*, *dark red trace*) show the locations used to measure the time course of the rhod-2 fluorescence. *Arrows* and *signs* under the traces indicate both the polarity and the time when the pulses where applied. Vertical scale: ΔF/F_0_ = 4; horizontal scale: time 200 ms

**Fig. 4:**
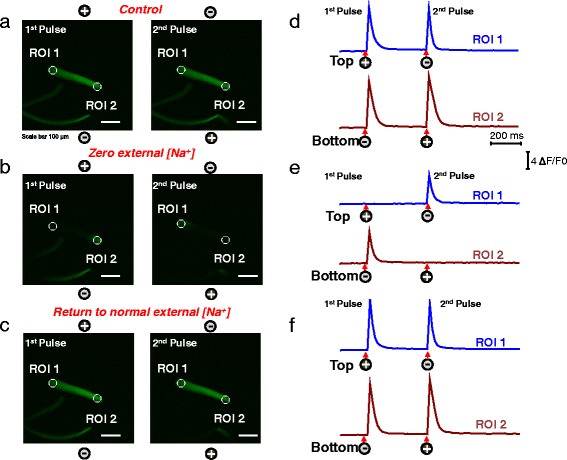
Myofibers with uniform responses challenged with a Na^+^-free extracellular solution exhibit local ALT Ca^2+^ transients that reverse to UNI responses upon return to normal Na^+^ containing external solution. Myofibers with uniform responses were loaded with rhod-2 and their Ca^2+^ transients elicited by electrical stimulation were recorded (**a**) before, during the treatment with a Na^+^-free external solution (**b**), and after washout and return to normal physiological solution (**c**). After challenging the myofiber with Na-free solution their Ca^2+^ transients show local and alternate responses when stimulated with alternating polarity bipolar field stimulation. Upon washout of the Na^+^ free solution and return to physiological conditions, the uniform behavior is completely restored. Rhod-2 signals were imaged and analyzed as in Fig. 1. See video in Additional file 5 (UNI myofiber challenged with Na^+^-free solution, and the reversibility of effects of Na^+^-free solution) for the entire time series. The *polarity signs* indicate the location of the electrodes and their polarity at any given time. *Scale bars* in **a–c** are 100 μm. **d–f** shows the time course of the rhod-2 fluorescence measured at the ends of the myofibers. *Circles* labeled as regions of interest (ROI) show ROI 1 (myofiber’s *upper end*, *blue trace*) and ROI 2 (myofiber’s *lower end*, *dark red trace*) the locations used to measure the time course of the rhod-2 fluorescence. *Arrows* and *signs* under the traces indicate both the polarity and the time when the pulses where applied. Vertical scale: ΔF/F_0_ = 4; horizontal scale: time 200 ms

The assessment of T-tubule morphology revealed no detectable morphological alterations in the ALT myofibers when compared to UNI counterparts. This finding suggests that the defect in ECC seen in ALT myofibers arises from dysfunction of ion channels (i.e., Na_v_1.4) involved in the generation and propagation of the action potential. To examine the contribution of Na^+^ channels, we first treated control myofibers with tetrodotoxin (TTX) and monitored electrically evoked Ca^2+^ transients. The addition of TTX converted a previously UNI myofiber (Fig. 3a) into an ALT myofiber (Fig. 3b; see video in Additional file 4). In the presence of TTX, Ca^2+^ signals were localized and restricted to the ends of the myofiber, and this effect was not reversible in a time window of 10 min (Fig. 3b). Similar observations were found when we perfused the myofibers with an extracellular physiological solution without Na^+^ (Na^+^-free solution, Fig. 4). Within minutes of Na^+^-free solution perfusion of the recording chamber, the reduction in extracellular Na^+^ concentration converted a previously UNI myofiber (Fig. 4a) into an ALT myofiber (Fig. 4b). In conditions of zero extracellular [Na^+^], the Ca^2+^ signals were localized and restricted to alternating ends of the myofiber during alternating polarity stimulation, and this effect was fully reversible (Fig. 4c; see video in Additional file 5). In another set of experiments, we removed Ca^2+^ from the recording solution and separately tested for the effects of nifedipine (20 μM; to block Ca^2+^ currents trough L-type Ca^2+^ channels [67]), a Ca^2+^ channel blocker (data not shown). The removal of Ca^2+^ or the addition of the Ca^2+^ channel blocker did not modify the properties of the electrically induced Ca^2+^ transients, implicating local TT voltage sensor-induced activation of coupled sarcoplasmic reticulum (SR) Ca^2+^ release channels.

### Reduction of extracellular [Cl^−^] does not modify the overall response of UNI or ALT myofibers

**Fig. 5:**
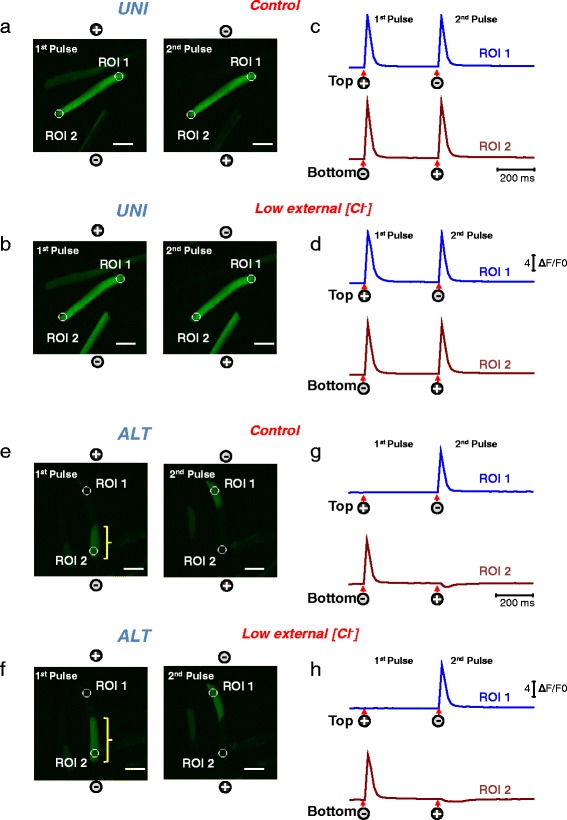
Myofibers with UNI or ALT responses challenged with a low [Cl^−^] extracellular solution exhibit modest alterations on excitability. Myofibers with uniform or alternate responses were loaded with rhod-2 and their Ca^2+^ responses to electrical stimulation were recorded before and after the perfusion with a low [Cl^−^] external solution. Spatio-temporal properties of electrically induced Ca^2+^ transients in myofibers that exhibited uniform or alternate responses in a physiological recording solution (**a** UNI, **e** ALT) and after treatment with a low [Cl^−^] external solution (**b** UNI, **f** ALT). Rhod-2 signals were imaged and analyzed as in Fig. 1. *Scale bars* in **a**, **b**, **e,** and **f** are 100 μm. UNI myofibers displayed a global Ca^2+^ transient in response to pulses of alternate polarity, and the perfusion of the low [Cl^−^] external solution produced negligible effects on the properties of the Ca^2+^ transients. Similarly, ALT myofibers displayed only local non-propagated responses at alternating ends upon application of pulses of alternate polarity and perfusion of the low [Cl^−^] external solution produced negligible effects on the overall ALT phenotype; however, in ALT fibers exposed to low [Cl^−^] solution, the longitudinal spread of the electrically elicited Ca^2+^ transient is increased (compare brackets length in **e** and **f**). **c**, **d**, **g,** and **h** show the time course of the rhod-2 fluorescence measured at the ends of the myofibers. *Circles* labeled as regions of interest ROI 1 (myofiber’s *upper end*, *blue trace*) and ROI 2 (myofiber’s *lower end*, *dark red trace*) show the locations used to measure the time course of the rhod-2 fluorescence. *Arrows* and *signs* under the traces indicate both the polarity and the time when the pulses were applied. Vertical scale: ΔF/F_0_ = 4; horizontal scale: time 200 ms

The above results implicate the capability of the fibers to generate an action potential as an important step in the establishment of the UNI or ALT responses and suggest that a reduced Na^+^ channel activity results in the generation of the ALT phenotype. These observations indicate that the Na^+^ channels contribute to this phenotype, but do not rule out the possible contribution of other channels such as K^+^ or Cl^−^ channels, which are also crucial for the excitability of the muscle fibers. Here, we tested whether Cl^−^ channels play a role in the determination of the ALT phenotype. To this end, we used a recording solution with a low Cl^−^ concentration (3 mM, low [Cl^−^]_out_). Figure 5 illustrates representative action potential-induced Ca^2+^ transients recorded in myofibers with UNI (Fig. 5a) or ALT (Fig. 5e) responses maintained in a control recording solution, or in a low [Cl^−^]_out_ recording solution (Fig. 5b; UNI fiber and Fig. 5f; ALT fiber).

Most of the UNI (94 %) or ALT (78 %) fibers exhibited little, if any, alterations in their electrically evoked Ca^2+^ responses when challenged with the low [Cl^−^]_out_ recording solution. In the case of UNI fibers, only 1 out of 18 fibers tested converted from UNI to ALT phenotype when challenged with the low [Cl^−^]_out_ solution. In the case of ALT fibers, only 4 out of 19 fibers converted from ALT to UNI when exposed to the low [Cl^−^]_out_ recording solution. In addition, only 4 out of 18 UNI fibers and 2 out of 19 ALT fibers exhibited signs of hyperexcitability (i.e., spontaneous repetitive twitching) when exposed to low [Cl^−^]out recording solution. Similar observations were made when the chloride channel blocker (9-anthracen carboxylic acid (9-AC) at 100 μM) was included in the control recording solution (data not shown). One interesting observation related to the low [Cl^−^]_out_ recording was made in the ALT fibers and the longitudinal spread of the electrically evoked Ca^2+^ transient. In these ALT fibers, a reduction of [Cl^−^] in the extracellular solution produced an extended spread of the Ca^2+^ transient from the fiber’s end towards the middle region of the fiber. This effect is indicated with the yellow brackets in Fig. 5ef and f. Note that the size of the spread is increased in low [Cl^−^]_out_ recording solution (Fig. 5f; yellow bracket) compared to normal [Cl^−^]_out_ solution (Fig. 5e). The average length of the Ca^2+^ spread from fiber’s end towards the middle of the fiber was significantly increased in the same fibers after challenge with low [Cl^−^]_out_ recording solution (average and SEM values were: 171 ± 21.9 μm in control solution; *192 ± 33 μm in low [Cl^−^]_out_, and 181.88 ± 30.9 μm in control solution plus 9-AC; *indicates *P* = 0.04408 vs. control, two-sample *t* test).

### Na_v_1.4 distribution evaluated by indirect immunofluorescence reveals no changes in Na^+^ channel distribution in ALT myofibers compared to UNI myofibers

**Fig. 6:**
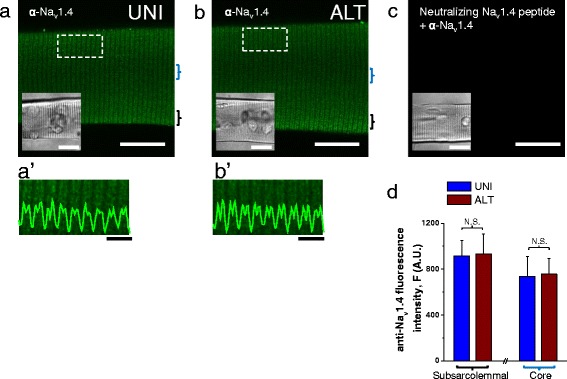
Na_v_1.4 sodium channel localization in UNI and ALT myofibers. Exemplar confocal images of UNI (**a**) and ALT (**b**) myofibers labeled with antibody to the intracellular II-III loop of Na_v_1.4. UNI and ALT fibers were identified using field stimulation and their locations within any tested dish were saved using a computer-controlled and motorized microscope stage, and then subjected to immunostaining protocol. After immunostaining, the same computer-controlled stage was used to localize and image the previously stored locations of corresponding UNI and ALT myofibers and then confocal imaging was performed. In each case, images show single confocal slices through the middle of the myofibers. *Scale bars* in **a–c** are 100 μm. **a'**–**b'** panels are zoomed-in versions of boxed regions indicated in panels **a** and **b**. Traces inserted on **a'**–**b'** are averaged fluorescence profiles across the box, *scale bars*: 4 μm. In both **a**-**a'** and **b**-**b'**, the immunofluorescent staining is localized to repeated transversely oriented bands whose general periodicity corresponded to that of T-tubules (see Fig. 2a, b). These images show that there are little, if any, differences in the immunolocalization of Na_v_1.4 channels between UNI and ALT myofiber types. **c** shows myofiber where the Na_v_1.4 antibody was preincubated with the Na_v_1.4 II-III loop neutralizing peptide before labeling. *Insets* in **a**-**c** are transmitted light images of myofibers shown in confocal images. **d** *bar plot* summarizing Na_v_1.4 channel staining intensity measured at the subsarcolemmal and core regions of UNI and ALT myofibers. *N.S.* Indicates *P* > 0.05 when compared with UNI control fibers, two-sample *t* test

Is the level of expression or localization of Na^+^ channels different in ALT myofibers compared to UNI myofibers? An alteration in the level or distribution of Na^+^ channels could explain the abnormalities seen in ALT myofibers. Using indirect immunofluorescence confocal microscopy and an α subunit specific Na_v_1.4 antibody, we observed the localization of voltage-gated Na^+^ channels in both UNI and ALT myofibers. In UNI myofibers, Na_v_1.4 was localized in a striated pattern (Fig. 6a). The Na_v_1.4 antibody clearly localized to structures organized in a repeated pattern within the labeled myofiber. Transversely oriented, regularly spaced bands of fluorescence were seen as two fluorescent lines separated by a thin unlabeled region, very similar to the staining of Na_v_1.4 previously reported [68]. The same localization (Fig. 6b) and staining intensity of Na_v_1.4 channels (Fig. 6d) that we observed in UNI myofibers was also seen in ALT myofibers, indicating that the overall localization and density of Na^+^ channels is similar between control and ALT myofibers. Thus the Na^+^ channels appear to be present, but not functioning normally in ALT fibers.

### Remote bipolar vs. focal unipolar electric field stimulation in ALT and UNI myofibers

Our above results show that uniform, full myofiber responses to bipolar field stimulation can be converted to non-propagated local depolarization-induced Ca^2+^ transients when Na^+^ channel conductance was compromised experimentally in such UNI myofibers. Similar local non-propagated depolarization-induced Ca^2+^ transients were observed in ALT myofibers without any experimental suppression of Na^+^ channel activity. These results allow us to hypothesize that ALT myofibers have dysfunctional Na^+^ channels and that depolarization-induced local responses are the result of direct local depolarization of the membrane and subsequent activation of Ca_v_1.1/RyR1 channel coupling and Ca^2+^ release. To test our hypothesis, we used electrical field stimulation via a small local unipolar electrode in the immediate vicinity of the myofiber, together with a remote reference electrode.

**Fig. 7:**
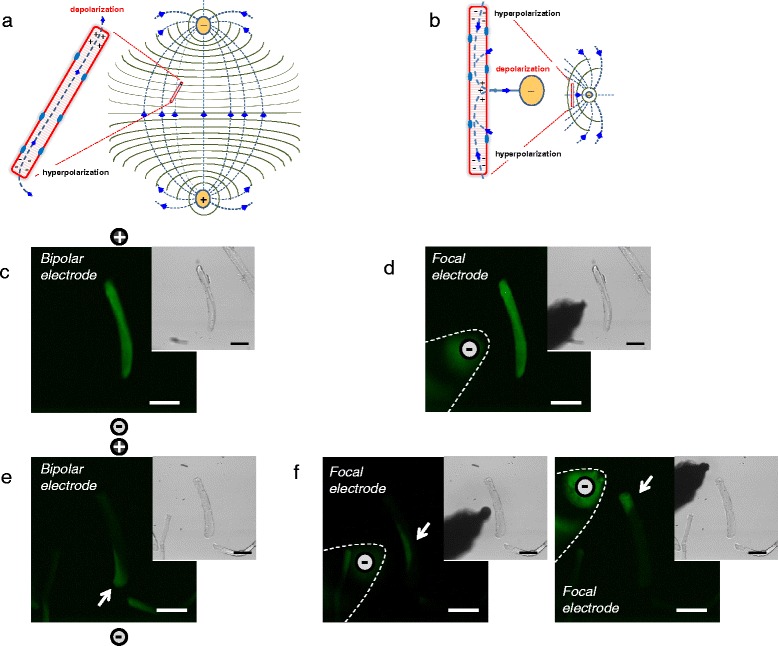
Extracellular electrode configurations used for electrical field stimulation of isolated myofibers and comparison of responses to stimuli by bipolar and local unipolar electrodes. **a** Theoretical dipole electrical field pattern and isopotential lines generated with two remote Platinum electrodes, separated by about 5 mm and oriented perpendicular to the bottom of the dish. **b** Theoretical unipolar electrical field pattern and isopotential lines generated with a focal tungsten extracellular stimulating electrode together with a remote field electrode (note: these *cartoons* represent a simplified approximation to the size, location, and orientation of the electrodes, electrical field, isopotential lines, and current fluxes). **c–f** Spatial properties of electrically induced Ca^2+^ transients in UNI and ALT myofibers in response to bipolar or focal unipolar stimulation. In each case, images are confocal snap shots of the time series at the peak of the transient in response to field stimulation (0.5 ms; 15 V/cm). **c** and **d** shows spatial properties of electrically induced Ca^2+^ transient of a UNI myofiber stimulated with a bipolar electrode (**c**) or with the focal electrode and using the same myofiber (**d)** (note: the spatial properties of the Ca^2+^ transients elicited by the bipolar or the unipolar electrode are similar in the UNI myofiber; the signals spread across the entire myofiber regardless of the electrode configuration used). **e, f** illustrates spatial properties of electrically induced Ca^2+^ transients of an ALT myofiber stimulated with bipolar electrodes (**e**) or with the focal electrode (**f**) either positioned near the center (**f** *left panel*) or near the upper end (**f** *right panel*) using the same myofiber. Note the differences in the spatial properties of the Ca^2+^ transients in the ALT myofiber when elicited with remote bipolar or local unipolar electrode. In the case of bipolar stimulation the Ca^2+^ transient is local and restricted to one end of the myofiber (**e**); however, when activated by unipolar stimulation, the Ca^2+^ signal occurs in the myofiber region in close proximity with the location electrode (**f**). *Insets* in **c–f** are transmitted light images to illustrate myofibers and electrode location

Depolarization-induced Ca^2+^ transients were measured and compared in the same myofibers using bipolar or unipolar field stimulation. Figure 7a illustrates the bipolar electrode configuration, the theoretical dipole electrical field pattern and isopotential lines. Figure 7b illustrates the local unipolar electrode configuration with remote reference electrode (not shown), and the theoretical dipole electrical field pattern and isopotential lines. Figure 7c shows a myofiber stimulated with a bipolar electrode, producing a propagated depolarization-induced uniform Ca^2+^ transient, which obscures the pattern of local electrotonic depolarization directly due to the stimulating pulse. A similar uniform response was observed in the same UNI myofiber when stimulated using the unipolar electrode (with negative polarity; Fig. 7d). In contrast, when using bipolar stimulation, local non-propagated depolarization-induced Ca^2+^ transients were observed at the ends of ALT myofibers (Fig. 7e, arrow). In addition, when the same ALT myofiber was stimulated with the cathodic unipolar electrode, positioned either near the center (Fig. 7f, left) or at one end of the myofiber (Fig. 7f, right), a local Ca^2+^ transient was now observed underneath the cathode where the myofiber membrane was depolarized (Fig. 7f, arrows). These results indicate that the local Ca^2+^ transients observed in ALT myofibers using either remote bipolar electrodes or a focal unipolar negative electrode result from direct activation of the Ca_v_1.1 via coupling to RyR1 voltage sensors in response to local partial depolarization of the sarcolemma near the working “depolarizing” electrode.

### Alternate Ca^2+^ responses occur in whole muscles of wild-type and MDX mice

**Fig. 8:**
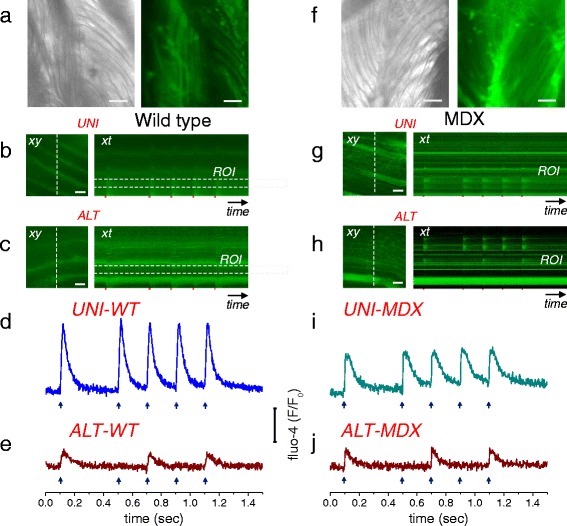
Alternate Ca^2+^ responses are observed in muscle fibers stimulated by bipolar field stimulation in whole muscle from wild-type and MDX mice. **a** *Left panel,* transmitted light image (×10 objective) of a segment of a FDB muscle isolated from wild-type mouse showing bundles of myofibers. *Right panel*, confocal image of the same FDB muscle segment shown on left panel, loaded fluo-4-AM. The whole muscle was explored in search of alternate responses using line-scan (*xt*) imaging (2 ms/line). **b** Representative confocal frame (*xy*, *left panel*) and line-scan images (*xt*, *right panel*) of a FDB segment illustrating uniform (UNI-WT) responses to a single field stimulus followed by a train of four pulses at 5 Hz. **c** representative frame (*xy*; *left panel*) and line-scan (*xt*; *right panel*) images of a FDB segment illustrating alternate (ALT-WT) responses to the same field stimulation pattern used in panel **b. b, c** were imaged with a × 63/1.5 NA objective. Trace in panel **d** shows time course of fluo-4 signals in the UNI-WT fiber (blue trace). Trace in panel **e** shows time course of fluo-4 signals of the ALT-WT fiber (*red trace*). **f** *Left panel,* transmitted light image (×10 objective) of a segment of a FDB muscle isolated from MDX mouse. *Right panel*, confocal image of FDB muscle segment, shown on *left panel*, loaded fluo-4-AM. **g** Exemplar confocal frame (*xy*, *left panel*) and line-scan images (*xt*, *right panel*) of a FDB segment from MDX mouse illustrating uniform (UNI-MDX) responses to field stimuli as used in panel **b. h** representative confocal frame (*xy*; *left panel*) and line-scan (*xt*; *right panel*) images of a FDB segment from MDX mouse illustrating alternate (ALT-MDX) responses to the same field stimulation pattern used in panel **b. g, h** were imaged with a × 10/0.3 NA objective. **i** shows time course of fluo-4 signals in the UNI-MDX fiber (*cyan trace*). **j** shows time course of fluo-4 signals of the ALT-MDX fiber (*red trace*). The *dashed rectangles* in **b**, **c**, **g,** and **h** (*right panels*) show regions of interest (ROI) used to measure the fluorescence time course. Note that the Ca^2+^ transients of the UNI-WT and UNI-MDX fibers (**d** and **i**) occur in response to each single stimulus (*arrows under traces*), whereas the ALT-WT and ALT-MDX fibers (**e** and **j**) respond to every other stimulus (see *arrows under traces*). *Scale bars* in **a**, **f–h**: 100 μm; **b** and **c**: 20 μm

The above results show that ALT myofibers are present in cultures of dissociated FDB fibers. How do ALT myofibers originate? Is the presence of ALT myofibers a consequence of the enzymatic dissociation and mechanical trituration required to isolate individual myofibers? If that is the case, Ca^2+^ signals from myofibers in undigested intact muscle should be devoid of ALT fibers. In order to evaluate this idea, we measured Ca^2+^ signals from individual myofibers present in whole FDB muscles that were previously dissected and then loaded with a membrane permeable Ca^2+^ indicator (Fig. 8a). To circumvent movement artifact, FDB muscles were held in place with a plastic coverslip, and the stimulation was performed by parallel electrodes glued to the dish lid (see Additional file 1: Figure S3). Twitch activity upon bipolar field stimulation was verified visually. The muscle was stimulated with a single electric pulse followed by a train of four pulses at 5 Hz with all the pulses alternating in polarity. This low-frequency protocol was preferred to further minimize movement artifacts related to electrical field stimulation. Then, the entire muscle was explored in search for uniform and alternate responses using line-scan (*xt*) imaging while simultaneously performing real-time analysis of the Ca^2+^ transients. In two out of ten muscles analyzed, we were able to identify alternate responses upon field stimulation of alternate polarity. Figure 8b illustrates exemplar line-scan profile (*right panel*) and Fig. 8d the corresponding Ca^2+^ transient time course from a UNI fiber using this stimulation protocol. Note that the Ca^2+^ transients of the UNI fiber shown in Fig. 8d occurs in response to each single stimulus (arrow under trace). On the other hand, Fig. 8c shows representative line-scan (*right panel*) image and Fig. 8e the corresponding Ca^2+^ transient time course, of a FDB fiber illustrating alternate (ALT) responses to field stimulation.

In another set of experiments, also using whole muscle Ca^2+^ imaging, we searched for the presence of ALT responses in muscles isolated from MDX mice, widely used as an animal model of Duchenne muscular dystrophy, the most common and severe muscular dystrophy. MDX muscles were explored and stimulated with the same protocol used in wild-type muscles. In four out of eight muscles analyzed (two FDB and six EDL), we were able to identify alternate responses upon field stimulation of alternate polarity. Figure 8g illustrates exemplar line-scan image Ca^2+^ signals (*right panel*) and Fig. 8i the corresponding Ca^2+^ transient time course from a UNI-MDX myofiber from a FDB muscle. Note that the Ca^2+^ transients of the UNI-MDX myofiber shown in Fig. 8i occur in response to each single stimulus in the train of alternating polarity stimuli. Figure 8h and i show representative line-scan image Ca^2+^ signals (*right panel*) and corresponding Ca^2+^ transients time course, respectively, of a MDX myofiber displaying alternate (ALT-MDX) responses to alternate polarity field stimulation. Remarkably, the Ca^2+^ transients of the ALT-WT (Fig. 8e) and ALT-MDX myofibers (Fig. 8i) only respond to every other stimulus, a demonstration that ALT behavior occurs in myofibers located in the intact (non-digested, non-triturated) whole muscles isolated from both wild-type and MDX mice.

## Discussion

Enzymatically dissociated adult skeletal muscle myofibers are routinely used in studies of muscle function such as the excitation-contraction coupling (ECC) [12, 13, 23, 26, 27, 64] and excitation-transcription coupling (ETC) processes [42, 47]. Here, we analyzed the contractile responses of individual myofibers to electric field stimulation using transmitted light microscopy, and monitored the spatial distribution of the Ca^2+^ transients using rhod-2, a non-ratiometric Ca^2+^ indicator, together with ultra-fast confocal microscopy. We show that the majority of skeletal muscle myofibers enzymatically dissociated and cultured for 1–2 days exhibit spatially uniform responses to electric field stimulation, and are thus in general useful for experimentation. In addition, we present for the first time a detailed study of a variable small fraction of myofibers that, despite normal gross morphology, exhibited abnormal ECC properties; they were unable to contract homogenously in response to electrical field stimuli and, most notably, exhibited local contractions at alternating myofiber ends in response to stimuli of alternating polarity during stimulation using remote bipolar electrodes. The Ca^2+^ transients were also modified in ALT myofibers, both temporally and spatially. The myofiber end exhibiting the local response alternated between ends when the polarity of the field stimulus from bipolar remote electrodes was alternated. This is a characteristic sign of a local, non-propagating membrane depolarization causing a local release of Ca^2+^, with the local depolarization alternating between ends when the direction of current flow is reversed when using remote bipolar electrodes. It is possible that ALT responses observed in cultured myofibers isolated from healthy muscles arise from mechanical damage during muscle dissection and or dissociation; however, ALT responses can also be detected in some myofibers from the intact (non-digested, non-triturated) isolated whole muscles, indicating that the enzymatic treatment is not required to produce the ALT response. The ALT behavior of myofibers from intact muscle was observed in 20 % of the preparations and thus is likely to be a rare event in healthy muscle. In muscles from the MDX mice, the ALT responses were observed more frequently (observed in 50 % of the preparations). It is anticipated that ALT responses could be also detected in different muscle disease models that affect excitable properties of myofibers.

The above changes in dissociated ALT myofibers were present in the absence of changes in resting Ca^2+^ or in resting membrane potential. Our data on resting Ca^2+^ is limited to global resting Ca^2+^, and one cannot rule out the possibility that changes in local Ca^2+^ domains, or microdomains [69–72], could contribute to the development of atypical excitability of ALT myofibers. Our resting membrane potential measurements were within the range of those previously reported for enzymatically dissociated FDB fibers [7, 62]. The similarity in resting membrane potential between ALT and UNI myofibers indicates that membrane depolarization does not account for the properties of the electrically induced Ca^2+^ transients seen in ALT myofibers.

The contractile ALT behavior in response to alternating field stimuli provides a simple and convenient screening procedure for detecting fibers with defective excitability, but with maintained voltage-dependent Ca^2+^ release. By simple microscope observation or time-lapse imaging of movement or of cytosolic Ca^2+^, it is possible to determine if one end of a fiber contracts with both polarity stimuli, or whether it responds only to pulses of a single polarity, but not to the other polarity. This immediately identifies fibers with defective excitability.

A few groups have investigated the impact of various electrical field stimulation configurations on skeletal muscle excitation [73–75]. It has been demonstrated that the effects of stimulation parameters (i.e., pulse strength and duration), electrode configuration (i.e., wire vs. plate electrodes) and electrode positioning (i.e., parallel vs. transverse, relative to the myofiber’s long axis) determines sites of excitation in a whole muscle preparation [73]. The patterns of passive myofiber polarization in response to electric field stimulation using alternative electrode geometries are different (Fig. 7). Electrical field stimulation via remote bipolar electrodes (Fig. 7a) results from the flow of current from a positive to a negative extracellular stimulating electrode, which produces an electric field in the interstitial space or recording chamber (volume conductor) [76–78]. When the electrical field is aligned (either perfectly or partially) with the long axis of the myofiber, the largest changes in membrane polarization occur with opposite polarity at the ends of the cell (Fig. 7a) [79, 80], with a continuous (and similar) change in both intracellular and extracellular voltage occurring along the myofiber, resulting in little transmembrane polarization occurring along the cell length [81, 82]. On the other hand, when a small unipolar field electrode (a single current-carrying conductor, comparable in diameter to a muscle myofiber and insulated up to near its tip) is placed in the vicinity of myofiber (Fig. 7b), current passes to or from the focal electrode (depending on its polarity, negative or positive), through the extracellular fluid surrounding the tissue of interest, and ultimately from or to a distant counter electrode depending on the electrode polarities. During cathodic stimulation, the negative charge of the focal electrode causes a redistribution of charge on the myofiber membrane, with negative charge collecting on the outside of the membrane underneath the cathode (depolarizing the membrane) (Fig. 7b). Associated with the depolarization of the membrane under the cathode is movement of positive charge intracellularly from the fiber’s ends to the region under the electrode, and hyperpolarization of the membrane at a distance away from the electrode. Note that for both focal cathodic stimulation and remote bipolar stimulation, if the myofiber is excitable and the local depolarization induced by the electric field stimulation reaches threshold, then the local stimulus will result in a propagated depolarization over the entire myofiber. Bipolar and unipolar electrode configurations have complex voltage and current patterns, and their numerical solutions have been reviewed in detail by others [83, 84].

Alternating (ALT) response myofibers exhibited no apparent gross morphological differences from the typical uniformly (UNI) responding myofibers, which contract uniformly and respond to both polarity electrical pulses. In the majority of ALT myofibers, di-8-ANEPPS staining shows little, if any, disruptions of the T-tubule network when compared to myofibers with uniform responses. However, the absence of gross changes in the di-8-ANEPPS staining does not rule out possible ultrastructural differences. Despite the apparent absence of alterations in the T-tubule network, the spatial characteristic of the Ca^2+^ transients were abnormal in the ALT myofibers, as indicated by their asymmetric contractility and Ca^2+^ transients. Our experiments with TTX and extracellular [Na^+^] removal, in which uniform myofibers are “converted” into ALT myofibers, suggest that Na^+^ channels contribute to the development of the ALT phenotype. Similar responses have been reported for whole skeletal muscle and skinned myofibers treated with TTX and stimulated with prolonged pulses [73, 85]. Our observations are not the result of TTX being incapable to block Nav1.4 channels. The same TTX concentration used here is enough to block skeletal muscle action potential and Na^+^ currents [52, 64], and in these myofibers, Nav1.5, the TTX-resistant channel, is a minor contributor to the Na^+^ channel population [86]. Furthermore, Na^+^ reduction would similarly suppress activation by Na_v_1.4 and 1.5. These data thus suggest that Ca^2+^ transients and local contractility in ALT fibers result from direct local depolarization of the Ca_v_.1.1 voltage sensor of ECC [73, 85].

The localization and function of the voltage-dependent sodium channels, Na_v_1.4, are critical for the initiation and propagation of the skeletal muscle action potential [87, 88]. Early work on the function and cellular localization of the Na_v_1.4 showed that these channels are expressed in both the sarcolemma and T-tubules with higher expression near the neuromuscular junction [89]. Our immunofluorescence approach indicates no detectable differences in the distribution and density of the Na_v_1.4 channels between UNI and ALT myofibers, suggesting that neither the location nor distribution may account for the alternate phenotype. We hypothesize that despite an apparently normal location and density of the Nav1.4 in ALT myofibers, changes in their function could be responsible for suppressed action potential generation. An interesting possibility may be the modification of gating parameters (i.e., slow inactivation) by modulation of β subunits [90].

Finally, we cannot conclude that the observed properties of ALT myofibers arise exclusively from the dysregulation of Na^+^ channels. In skeletal muscle, the action potential depolarization is mediated by the opening of voltage-gated Na^+^ channels, and the repolarizing phase is due in part to the inactivation of the voltage-gated Na^+^ channels and the opening of multiple K^+^ channels (both voltage-dependent and independent), as well as contributions from Cl^−^ channels [91, 92]. Given the importance of Cl^−^ channels in skeletal muscle excitability [93] we investigated whether Cl^−^ channels have a role in the establishment of the ALT phenotype. Our experiments with acute (10–30 min) reduction of [Cl^−^] in the bath solution indicate that this manipulation negligibly affects the excitation properties of UNI myofibers (i.e., reduced [Cl^−^] promotes conversion of UNI to ALT in less than 6 % of the fibers examined) in response to alternating field stimulation. The reduction of [Cl^−^] influences the excitable properties in a small percentage the ALT fibers (i.e., causes ALT to UNI conversion in less than 21 % of the fibers). Also, less than 20 % of UNI and ALT fibers displayed spontaneous hyperexcitability in reduced [Cl^−^] solution. Another interesting observation was that the reduction of [Cl^−^] in ALT fibers results in an increased spread of the local Ca^2+^ transient in response to field stimulation when compared to the response elicited in control solution. This result indicates that Cl^−^ channels regulate the fiber’s electrical length constant. Overall, our results indicate that Cl^−^ channels may contribute, but are not critical, to the manifestation of the ALT behavior in response to field stimulation. Whether other ion channels important for skeletal muscle action potential could play a role in the suppressed excitation behavior of the ALT myofibers remains to be determined.

## Conclusions

Although it is clear that ALT myofibers can be present in FDB whole muscle and FDB cultures, many aspects of its origin are still being investigated. Are ALT myofibers present in vivo? Do ALT myofibers influence or underlie disease conditions? Regardless of the mechanism(s) responsible for abnormal excitability of ALT myofibers, we encourage the careful monitoring of the contractile response of the myofiber (and if available, the evaluation of the Ca^2+^ transients) to establish that normal behavior of skeletal muscle myofibers be implemented when choosing myofibers for physiological experiments, and recommend avoiding the use of muscle myofibers that display only alternating end local contractile activity in response to alternating polarity stimuli (unless that is the objective). On the other hand, if defective myofiber excitability is suspected under various experimental or pathological conditions, our results suggest that looking for myofibers exhibiting ALT behavior in response to alternating polarity electric field stimulation via remote bipolar electrodes can provide a useful approach for identifying myofibers with compromised excitability.

## Additional files

Additional file 1: Figure S1. ALT fibers exhibit graded responses to pulses of increased duration and amplitude. Representative Ca^2+^ transients elicited by bipolar field suprathreshold pulses of fixed amplitude but increasing duration (0.5–5ms) measured in UNI myofiber (A) and at the responding end of the ALT myofiber (B). Signals were recorded using high-speed line scanning (100μs/line) to further characterize the temporal properties of the Ca^2+^ transients. Time course of the normalized Ca^2+^ transients in the UNI (C) and ALT (D) myofibers derived from panels A and B, respectively to compare the kinetics of Ca^2+^ transients. UNI fibers showed reproducible responses that were independent of the pulse duration, whereas ALT fibers exhibited graded and reproducible responses with duration proportional to the duration of the stimulus. E, Representative isochronical confocal *xy* images acquired milliseconds before stimulation and showing the spatial properties of the electrically evoked rhod-2 signals using monopolar field stimulation. Ca^2+^ transients were elicited by suprathreshold pulses of fixed duration (1 ms) but increasing amplitude (30–60 V) at one end of an ALT myofiber. Dashed lines are fixed in the horizontal axis in all frames and indicate the fiber’s end. The arrows mark the limit of longitudinal propagation of the Ca^2+^ signals. The increase in the gap between the dashed line (fiber’s end) and the arrow (Ca^2+^ front) with pulses of increased amplitude confirms the graded nature of these transients. Using this electrode configuration, this local Ca^2+^ signal appears to originate at the fiber’s end, spreads inwardly towards the fiber’s trunk and then suddenly stops without full propagation, which is opposite to the responses seen in UNI fibers (Fig. 6c, d). F, Shows the transmitted light image of the ALT myofiber and the location of the electrode used in panel E. Scale bars in E and F, 100 μm. **Figure S2.** ALT fibers have no differences in resting membrane potential when compared to UNI fibers. Resting membrane potential (RMP) was measured in L-15 media supplemented with 20 μM BTS (N-benzyl-p-toluene sulphonamide) to minimize movements during microelectrode penetration. The phenotype (UNI or ALT) was characterized by monitoring twitch activity using an inverted microscope (×10 magnification) and 1-ms suprathreshold bipolar field stimulation. A, Distribution of RMP values and fraction of fibers population in UNI and ALT groups. B, Bar plot summary. Mean RMP upon impalement in UNI (*n* = 35, *N* = 3) and ALT fibers (*n* = 24, *N* = 3) were −62.7 ± 0.9 and −59.2 ± 1.6, respectively. *N* indicates number of mice per condition, and *n* indicates number of fibers tested. ALT fibers showed no statistically significant difference in RMP when compared to UNI myofibers (two-sample *t* test, *P* > 0.05). **Figure S3.** Montage of whole muscle for electrical stimulation and Ca^2+^ imaging. After loading for 30 min with Fluo4-AM (20 μM), the muscle was immobilized in a holder chamber and then field stimulated. The muscles (*flexor digitorum brevis* or *extensor digitorum longus*) were carefully dissected, avoiding unnecessary manipulation of muscle tissue, such as excessive pulling, and keeping tendons at both ends, then muscles were briefly placed on the surface of a glass-bottomed petri dish (A). Silicone grease (high-vacuum grease, Dow Corning Corp, Midland, MI) was applied to the plastic surface of the petri dish (arrows). After applying the grease a small piece (14 × 8 × 0.5 mm) of a plastic cover slip (B–C; arrowheads) was placed on top of muscles and used to gently hold muscles in place during stimulation. The plastic cover slip was secured via the contact with the vacuum grease applied to the petri dish surface (B–C). Muscles were stimulated, using platinum electrodes attached to the lid of the petri dish (D, dashed lines). (DOCX 1633 kb)

Additional file 2: **Uniform and alternate electrically induced twitch responses.** Description: left, uniform electrically induced twitch responses. Representative transmitted light movie of a skeletal muscle myofiber illustrating its uniform twitch behavior in response to bipolar electrical field stimulation of alternate polarity. Right, alternate electrically induced twitch responses. Representative transmitted light movie of a skeletal muscle myofiber showing its alternate twitch behavior in response to bipolar electrical field stimulation. Twitch responses to a pair of pulses (1 ms; 15 V/cm) interspaced by a 400-ms interval. High-speed time-lapse imaging was conducted for 2 s at 16.7 ms/frame using a ×10 objective magnification. (AVI 5445 kb)

Additional file 3: **Uniform and alternate electrically induced Ca2+ transients.** Description: left, uniform electrically induced Ca^2+^ transients. Representative confocal time series of a skeletal muscle myofiber loaded with the Ca^2+^ indicator rhod-2 illustrating uniform Ca^2+^ transients in response to bipolar electrical field stimulation of alternate polarity. Right, alternate electrically induced Ca^2+^ transients. Representative confocal time series of a skeletal muscle myofiber loaded with the Ca^2+^ indicator rhod-2 showing its local and asymmetrical Ca^2+^ transients in response to bipolar electrical field stimulation of alternate polarity. Ca^2+^ transients were elicited by a pair of pulses (1 ms; 15V/cm) interspaced by a 400-ms interval. High-speed time-lapse imaging was conducted for 2 s at 16.7 ms/frame using a ×10 objective magnification. (AVI 2965 kb)

Additional file 4: **TTX treatment “converts” UNI myofibers to ALT myofibers.** Description: left, pre-TTX: uniform electrically induced Ca^2+^ transients. Confocal time series of a skeletal muscle myofiber loaded with the Ca^2+^ indicator rhod-2 illustrating uniform Ca^2+^ transients in response to bipolar electrical field stimulation of alternate polarity and before the addition of tetrodotoxin (TTX). Right, post-TTX: alternate electrically induced Ca^2+^ transients. Confocal time series of same myofiber presented in left panel, illustrating asymmetric Ca^2+^ transients in response to bipolar electrical field stimulation of alternate polarity 5 min after the addition of TTX; 1μM. Ca^2+^ transients were elicited by a pair of pulses (1 ms; 15V/cm) interspaced by a 400-ms interval. High-speed time-lapse imaging was conducted for 2 s at 16.7 ms/frame using a ×10 objective magnification. (AVI 3650 kb)

Additional file 5: **Reduction of extracellular [Na+] “converts” UNI myofibers to ALT myofibers.** Description: left, uniform electrically induced Ca^2+^ transients measured in physiological extracellular Na + concentration, [Na^+^]e. Confocal time series of a skeletal muscle myofiber loaded with the Ca^2+^ indicator rhod-2 illustrating uniform Ca^2+^ transients in response to bipolar electrical field stimulation of alternate polarity and before the addition of Na-free recording solution. Center, alternate electrically induced Ca^2+^ transients measured in Na^+^-free extracellular conditions. Confocal time series of the same myofiber displayed in left panel, illustrating asymmetric Ca^2+^ transients in response to bipolar electrical field stimulation of alternate polarity 5 min after the addition of the addition of Na^+^-free recording solution. Right, uniform electrically induced Ca^2+^ transients measured when myofiber is returned to recording solution with physiological extracellular [Na^+^]. Confocal time series of same myofiber displayed in center panel, illustrating uniform Ca^2+^ transients in response to bipolar electrical field stimulation of alternate polarity 5 min after the return to control solution. Ca^2+^ transients were elicited by a pair of pulses (1 ms; 15V/cm) interspaced by a 400-ms interval. High-speed time-lapse imaging was conducted for 2 s at 16.7 ms/frame using a ×10 objective magnification. (AVI 4415 kb)

## Abbreviations

ALT: alternate response
BSA: bovine serum albumin
Ca_v_1.1: voltage-dependent Ca^2+^ channel skeletal muscle isoform 1.1
di-8-ANEPPS: pyridinium, 4-[2-(6-(dioctylamino)-2-naphthalenyl) ethenyl]-1-(3-sulfopropyl)
ECC: excitation-contraction coupling
EDL: extensor digitorum longus
ETC: excitation-transcription coupling
FDB: flexor digitorum brevis
HEPES: 4-(2-Hydroxyethyl)piperazine-1-ethanesulfonic acid
LSM: laser scanning microscope
L-15: Leibovitz’s L-15 Media
MEM: minimum essential media
Na_v_1.4: voltage-dependent Na^+^ channel skeletal muscle isoform 1.4
NMDG: *N*-Methyl-D-glucamine
PBS: phosphate-buffered saline
RMP: resting membrane potential
ROIs: regions of interest
RyR1: ryanodine receptor type 1
SR: sarcoplasmic reticulum
TT: transverse tubules
TTX: tetrodotoxin
UNI: uniform response
