# Supplementary material for: Alternating bipolar field stimulation identifies muscle fibers with defective excitability but maintained local Ca2+ signals and contraction
Source: Skelet Muscle. 2016 Feb 5;6:6. doi: 10.1186/s13395-016-0076-8 (PMC4743112; doi:10.1186/s13395-016-0076-8)
Supplement: Supplementary file 1 — ALT fibers exhibit graded responses to pulses of increased duration and amplitude. Representative Ca2+ transients elicited by bipolar field suprathreshold pulses of fixed amplitude but increasing duration (0.5–5ms) measured in UNI myofiber (A) and at the responding end of the ALT myofiber (B). Signals were recorded using high-speed line scanning (100μs/line) to further characterize the temporal properties of the Ca2+ transients. Time course of the normalized Ca2+ transients in the UNI (C) and ALT (D) myofibers derived from panels A and B, respectively to compare the kinetics of Ca2+ transients. UNI fibers showed reproducible responses that were independent of the pulse duration, whereas ALT fibers exhibited graded and reproducible responses with duration proportional to the duration of the stimulus. E, Representative isochronical confocal xy images acquired milliseconds before stimulation and showing the spatial properties of the electrically evoked rhod-2 signals using monopolar field stimulation. Ca2+ transients were elicited by suprathreshold pulses of fixed duration (1 ms) but increasing amplitude (30–60 V) at one end of an ALT myofiber. Dashed lines are fixed in the horizontal axis in all frames and indicate the fiber’s end. The arrows mark the limit of longitudinal propagation of the Ca2+ signals. The increase in the gap between the dashed line (fiber’s end) and the arrow (Ca2+ front) with pulses of increased amplitude confirms the graded nature of these transients. Using this electrode configuration, this local Ca2+ signal appears to originate at the fiber’s end, spreads inwardly towards the fiber’s trunk and then suddenly stops without full propagation, which is opposite to the responses seen in UNI fibers (Fig. 6c, d). F, Shows the transmitted light image of the ALT myofiber and the location of the electrode used in panel E. Scale bars in E and F, 100 μm. Figure S2. ALT fibers have no differences in resting membrane potential when compared to UNI fibers. Re [file 13395_2016_76_MOESM1_ESM.docx]

**Alternating bipolar field stimulation identifies muscle fibers with defective excitability but maintained local Ca^2+^ signals and contraction**

Erick O. Hernández-Ochoa^1^, Camilo Vanegas^1^, Shama R. Iyer^2^, Richard M. Lovering^2^ and Martin F. Schneider^1^*

^1^Department of Biochemistry and Molecular Biology, University of Maryland School of Medicine, Baltimore, MD 21201

**^2^**Department of Orthopaedics, University of Maryland School of Medicine, Baltimore, MD 21201

**Additional file 1**

**Additional Figures 1-3**

**Additional Figure 1. ALT fibers exhibit graded responses to pulses of increased duration and amplitude.** Representative Ca^2+^ elicited by bipolar field suprathreshold pulses of fixed amplitude (applied at time 0) but increasing duration (0.5-5ms) measured in UNI myofiber (A) and at the responding end of the ALT myofiber (B). Signals were recorded using high speed line scanning (100µs/line) to further characterize the temporal properties of the Ca^2+^ transients. Time course of the normalized Ca^2+^ transients in the UNI (C) and ALT (D) myofibers derived from panels A and B, respectively to compare the kinetics of Ca^2+^ transients. UNI fibers showed reproducible responses that were independent of the pulse duration, whereas ALT fibers exhibited graded and reproducible responses with duration proportional to the duration of the stimulus. E, Representative isochronal confocal *xy* images acquired milliseconds before stimulation and showing the spatial properties of the electrically-evoked rhod-2 signals using monopolar field stimulation. Ca^2+^ transients were elicited by suprathreshold pulses of fixed duration (1 ms) but increasing amplitude (30-60 V) at one end of an ALT myofiber. Dashed lines are fixed in the horizontal axis in all frames and indicate the fiber’s end. The arrows mark the limit of longitudinal propagation of the Ca^2+^ signals. The increase in the gap between the dashed line (fiber’s end) and the arrow (Ca^2+^ front) with pulses of increased amplitude confirms the graded nature of these transients. Using this electrode configuration, this local Ca^2+^ signal appears to originate at the fiber’s end, spreads inwardly towards the fiber’s trunk and then suddenly stops without full propagation, which is opposite to the responses seen in UNI fibers (Fig. 7C-D). (F), Shows the transmitted light image of the ALT myofiber and the location of the monopolar electrode used in panel E. Scale bars in E and F, 100 µm.

**Additional Figure 2. ALT fibers have no differences in resting membrane potential when compared to UNI fibers.** Resting membrane potential (RMP) was measured in L-15 media supplemented with 20 µM BTS (N-benzyl-p-toluene sulphonamide) to minimize movements during microelectrode penetration. The phenotype was characterized by monitoring twitch activity using an inverted microscope (10x magnification) and 1 ms suprathreshold bipolar field stimulation. A, Distribution of RMP values and fraction of fibers population in UNI and ALT groups. B, Bar plot summary. Mean RMP upon impalement in UNI (n=35, N=3) and ALT fibers (n=24, N=3) were -62.7 ± 0.9 and -59.2 ± 1.6, respectively. *N*, indicates number of mice per condition and *n* indicates number of fibers tested. ALT fibers showed no statistically significant difference in RMP when compared to UNI myofibers (Two sample t Test, P>0.05).

**Additional Figure 3. Montage of whole muscle for electrical stimulation and Ca^2+^ imaging.** After loading for 30 min with fluo4-AM (20 µM), the muscle was immobilized in a holder chamber and then field stimulated. The muscles (*flexor digitorum brevis* or *extensor digitorum longus*) were carefully dissected, avoiding unnecessary manipulation of muscle tissue, such as excessive pulling, and keeping tendons at both ends, then muscles were briefly placed on the surface of a glass-bottomed petri dish (A). Silicone grease (high vacuum grease, Dow Corning Corp, Midland, MI) was applied to the plastic surface of the petri dish (arrows). After applying the grease, a small piece (14 mm x 8 mm x 0.5 mm) of a plastic cover slip (B-C; arrowheads) was placed on top of muscles and used to gently hold muscles in place during stimulation. The plastic cover slip was secured via the contact with the vacuum grease applied to the petri dish surface (B-C). Muscles were stimulated, using platinum electrodes attached to the lid of the petri dish (D, dashed lines).
